# WTe_2_ Weyl semimetal conical microarray enabled quantum-enhanced terahertz photocurrent sensing for organophosphorus toxin detection in simulated body fluid

**DOI:** 10.3389/fphys.2026.1997758

**Published:** 2026-10-13

**Authors:** Xu Zhang, Mingxin Li, Chunting Wu

**Affiliations:** 1 College of Criminal Science and Technology, Jilin Police College, Changchun, China; 2 School of Physics, Changchun University of Science and Technology, Changchun, China

**Keywords:** conical microarray, current responsivity, noise-equivalent power, nonlinear photocurrent rectification, organophosphorus pesticide, quantum-enhanced terahertz sensing, topological fermi arc states, Weyl semimetal WTe2

## Abstract

As a representative topological quantum system, type-II Weyl semimetals break the fundamental bandgap limitation of conventional semiconductor photodetectors and deliver giant intrinsic nonlinear optical response originating from topologically protected Weyl points and Fermi arc surface states, providing a disruptive material platform for advanced room-temperature terahertz (THz) optoelectronics. Building on this quantum material advantage, we develop a room-temperature THz sensor based on a periodic WTe_2_ conical microarray for quantitative detection of the organophosphorus neurotoxin chlorpyrifos in simulated body fluid (SBF). Each microcone measures 80 µm in base diameter and 60 µm in height, arranged with a 100 µm center-to-center pitch on a Si/SiO_2_ substrate with Au/Ti electrodes. A 140 GHz continuous-wave THz source illuminates the chip from above, and the photocurrent is read out by a Keithley 2400 digital source meter via two tungsten-carbide microprobes. Leveraging the geometric near-field concentration effect at the cone tips, the proposed architecture synergistically enhances the second-order nonlinear photocurrent rectification originating from the Weyl-point band structure of WTe_2_, as confirmed by CST electromagnetic simulation. The underlying sensing mechanism relies on chlorpyrifos-induced dielectric screening and interfacial charge transfer at the WTe_2_ surface, which modulate the THz-to-DC rectification efficiency. Current responsivity (Ra) and noise-equivalent power (NEP) are extracted from illuminated and dark current–voltage (I–V) characteristics as a function of chlorpyrifos concentration over a bias range of 7.0–8.0 V. The sensor achieves a limit of detection (LOD) of 12 ng/mL across a dynamic range of 0.05–100 µg/mL in 0.7% saline SBF, with a logarithmic calibration fit of R^2^ = 0.991. Compared with conventional semiconductor and thermal THz detectors, the topological-quantum-material device realizes room-temperature high-sensitivity detection without relying on interband transition or cryogenic cooling, demonstrating fundamentally superior performance stability and broadband adaptability. This work demonstrates that microstructured Weyl semimetal heterostructures enable quantum-enhanced THz biochemical sensing, providing a new device architecture for label-free, reagent-free toxin detection in complex aqueous environments.

## 1 Introduction

The rapid development of topological quantum materials has opened a new paradigm for designing high-performance next-generation optoelectronic devices, particularly in the terahertz (THz) frequency regime. As a representative type-II Weyl semimetal, tungsten ditelluride (WTe_2_) exhibits exceptional physical properties including broken inversion symmetry, ultrahigh carrier mobility, topologically protected surface states and giant second-order nonlinear optical response, making it an ideal active medium for room-temperature THz quantum photodetectors and sensors (Ali et al., 2014; Wang et al., 2023; Wang et al., 2025). Notably, the 140 GHz operating frequency lies in the millimeter-wave/low-THz transition region, and the nonlinear rectification physics and sensing mechanism demonstrated here are broadly applicable across the wider THz spectrum. Different from conventional semiconductor photodetectors that are inherently limited by bandgap constraints, Weyl semimetals enable broadband THz detection via nonlinear photocurrent rectification directly at room temperature, with performance governed by the topological band structure rather than impurity doping levels. Thanks to this intrinsic quantum-originated response mechanism, Weyl semimetal detectors deliver both fundamentally higher operation speed and better long-term stability than traditional thermal or photoconductive THz detectors, stimulating intense research interest in topological-material-based advanced THz technologies.

From a device performance perspective, traditional THz detection technologies all face inherent bottlenecks that are difficult to break through under the classical framework. Thermal detectors such as pyroelectric and bolometer devices suffer from slow response speed and susceptibility to ambient temperature drift, and cannot meet the demand for high-speed real-time sensing. Conventional semiconductor photoconductive detectors and quantum well detectors require interband or intersubband transitions to generate photocurrent, which are inherently limited by material bandgaps, usually require cryogenic cooling to achieve high sensitivity, and have narrow effective response bands. Plasmon-enhanced metal metasurface detectors rely on the ohmic loss of free electrons in noble metals, which have large intrinsic loss and low photoelectric conversion efficiency. Recent studies have demonstrated that integrating semimetal active materials with plasmonic architectures can effectively alleviate the intrinsic loss limitation and realize remarkable improvement in detection responsivity, offering a feasible route to break through the performance ceiling of conventional plasmonic THz detectors (Su et al., 2025). In sharp contrast, Weyl semimetal THz detectors based on topological quantum effects break through the above limitations from the physical mechanism: the second-order nonlinear rectification effect originating from Weyl points has no bandgap threshold, supports ultra-broadband response from microwave to far-infrared, works stably at room temperature, and has ultra-high carrier mobility and fast response speed, showing fundamental performance advantages that classical material systems cannot match.

While topological Weyl semimetals have shown remarkable potential in THz device physics, their application in biochemical sensing scenarios remains largely underexplored, especially for trace toxin detection in complex aqueous matrices. Organophosphorus (OP) compounds, including widely used pesticides such as chlorpyrifos (C_9_H_11_Cl_3_NO_3_PS), are among the most toxic agrochemicals in global use. Their accumulation in biological fluids poses severe health risks through irreversible inhibition of acetylcholinesterase (AChE), an enzyme essential for neurotransmission (Kalenik et al., 2026). Chlorpyrifos exposure is associated with acute neurotoxicity, developmental disorders, and long-term immunosuppression. Regulatory agencies including the World Health Organization (WHO) and Environmental Protection Agency (EPA) have established maximum residue limits (MRLs) of 10–50 ng/mL in blood plasma, motivating demand for rapid, sensitive analytical tools applicable to biological matrices (World Health Organization, 2017).

Conventional confirmatory analytical methods, including gas chromatography–mass spectrometry (GC–MS) and enzyme-linked immunosorbent assay (ELISA), provide high sensitivity and quantitative accuracy but require bulky laboratory instrumentation, time-consuming sample preparation and trained personnel, making them unsuitable for on-site rapid screening (Bhattu et al., 2021). Electrochemical biosensors based on AChE inhibition have emerged as promising point-of-care alternatives; however, their practical application is hindered by poor anti-interference ability in complex biological fluids and the inherent instability of enzyme reagents (Liu et al., 2025). Accordingly, there remains a pressing need for label-free, reagent-free detection platforms that combine high sensitivity with operational simplicity even in high-loss aqueous environments.

THz time-domain spectroscopy (THz-TDS) has attracted substantial interest for biochemical sensing owing to its non-ionizing nature and exquisite sensitivity to intermolecular interactions and low-frequency vibrational modes (Tonouchi, 2007). In-depth studies on THz vibrational spectra of small-molecule analytes and chemometric quantitative methods have further laid a methodological foundation for trace detection in complex aqueous matrices (Tu et al., 2023; Song et al., 2025). Nevertheless, direct THz transmission measurement of aqueous samples faces a fundamental bottleneck: water exhibits an absorption coefficient exceeding 150 cm^-1^ at 1 THz, which severely attenuates the incident THz field and degrades detection sensitivity (Liebe et al., 1991). To overcome this limitation, THz metasurfaces—engineered arrays of subwavelength resonant structures—have been developed to amplify the local electromagnetic field by orders of magnitude via localized surface plasmon resonance, dramatically increasing the interaction cross-section between the THz wave and the analyte (Bi et al., 2023; Agarwal et al., 2025). Substantial research progress in structural design and functional expansion has been systematically summarized in recent reviews, further promoting the development of THz metasurface biosensing toward practical applications (Zhu et al., 2024). In parallel, advances in THz functional devices such as polarization-sensitive modulators and high-sensitivity thin-film sensors have further expanded the application scope of THz technology to 6G communication and remote environmental monitoring (Song et al., 2023b; Song et al., 2023c; Song et al., 2024).

Although planar metasurfaces effectively enhance THz light-matter interaction, their performance is ultimately constrained by the limited interaction volume of two-dimensional thin films. Integrating topological quantum materials with three-dimensional microstructured photonic architectures represents a promising route to further boost THz detector performance beyond the limits of planar devices. Three-dimensional microstructured transducers provide a powerful extension of planar detector geometries by offering additional degrees of freedom for THz field coupling and photoelectric readout (Song et al., 2022; Xu et al., 2026). In particular, conical microarrays—featuring a progressive reduction in cross-sectional area from base to apex—have been shown to provide efficient near-field concentration via geometric focusing, analogous to the operation of a tapered waveguide or horn antenna (Liu et al., 2014; Xu et al., 2026). Thanks to this progressive taper design, the incident THz field is gradually compressed as it propagates from the wide base to the narrow apex, producing a localized electric field enhancement that amplifies the generated photocurrent per unit incident power without increasing the dark current. To date, a variety of 3D microstructured architectures have been demonstrated for THz detection, including micro-fold line arrays based on crystalline Weyl semimetal with Au nanofilms (Song et al., 2023d), laser-magnetic co-modulated plasmonic microstructures (Sun et al., 2025), microgroove arrays on phase-change alloy nanofilms (Song et al., 2023a), and 3D plasmonic confinement-enhanced hybrid detectors (Sun et al., 2026a; Wang et al., 2026). Notably, most of these prior 3D Weyl semimetal THz detector studies focused on pure optoelectronic performance characterization, whereas the present work specifically targets biochemical toxin sensing applications in complex aqueous matrices.

As a core active material for such 3D devices, WTe_2_ offers not only excellent nonlinear photoresponse but also intrinsic surface sensitivity suitable for sensing applications. As a type-II Weyl semimetal, WTe_2_ possesses a large second-order nonlinear photocurrent response arising from its broken inversion symmetry, and ultrahigh carrier mobility enabling efficient THz nonlinear rectification into a measurable DC photocurrent (Wang et al., 2023; Wang et al., 2025). Moreover, its layered crystalline structure with exposed chalcogenide edges provides abundant adsorption sites for analyte molecules, and its surface Fermi level is highly sensitive to the local electrostatic environment. This means the nonlinear conductivity of WTe_2_ can be directly modulated by adsorbate-induced charge transfer, enabling direct THz-to-DC photocurrent sensing without requiring an additional plasmonic metal layer. Beyond WTe_2_, other topological materials such as MnBi_2_Te_4_ have also achieved high-responsivity room-temperature THz detection (Sun et al., 2026b), while Weyl semimetal metasurfaces have been applied to nondestructive antibiotic detection (Hu et al., 2025b), demonstrating the broad application potential of this material system.

Building on the above advances, this work exploits the synergy between Weyl semimetal quantum properties and three-dimensional geometric field enhancement to realize a quantum-enhanced THz photocurrent sensing device for trace organophosphorus toxin detection. Using chlorpyrifos as the target analyte and 0.7% saline simulated body fluid (SBF) as the matrix, we integrate a WTe_2_ thin film with a 3D-printed conical microarray to achieve label-free, reagent-free detection at 140 GHz. Each cone has a base diameter of 80 µm, height of 60 µm, and center-to-center pitch of 100 µm. A Keithley 2400 digital source meter, connected via two tungsten-carbide microprobes, records the illuminated and dark I–V characteristics from which current responsivity Ra and noise-equivalent power (NEP) are directly extracted. The sensing performance is systematically characterized through three complementary modalities: DC photoelectric parameter analysis, THz-TDS verification, and chemometric quantitative evaluation. CST electromagnetic simulations verify the near-field concentration effect, and comprehensive material characterizations confirm high-quality WTe_2_ crystalline growth. The device achieves a 12 ng/mL limit of detection with fully label-free, enzyme-free operation, demonstrating the great potential of topological quantum materials for advanced THz biochemical sensing.

## 2 Device design, sensing principle, and fabrication

### 2.1 WTe_2_ conical microarray architecture

The core functional element of the proposed sensor is a periodically arranged WTe_2_ conical microarray, which is specifically designed to achieve simultaneous THz near-field concentration and efficient photocurrent readout (**Figure 1a**). The geometric parameters of each cone are: base diameter D_base = 80 µm, vertical height H = 60 µm, and center-to-center pitch P = 100 µm. To enable electrical readout, Au/Ti electrodes are deposited at the cone base and apex regions, defining a two-terminal lateral device with the THz-induced photocurrent traversing the full cone height along the vertical WTe_2_ channel. Notably, the 100 µm pitch is designed to be subwavelength relative to the free-space wavelength at 140 GHz (λ ≈ 2.14 mm), which ensures that the entire array operates as a collective electromagnetic absorber rather than a set of discrete scattering units.

**Figure 1:**
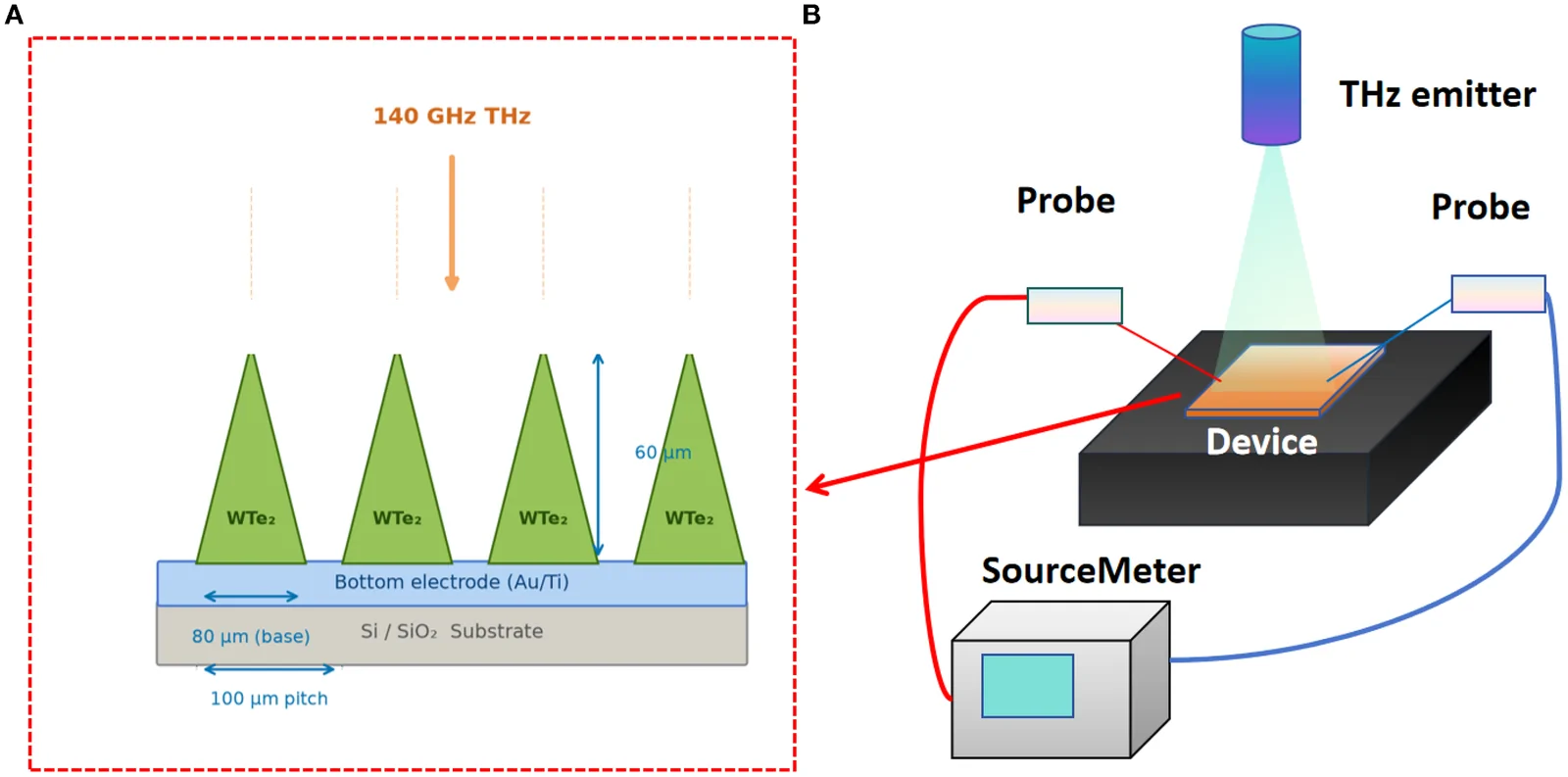
Conical microarray THz detector structure and experimental setup. **(a)** Cross-sectional schematic of the WTe_2_ conical microarray (80 µm base diameter, 60 µm height, 100 µm pitch) on Si/SiO_2_ substrate with Au/Ti electrodes (Ti 5 nm/Au 80 nm); 140 GHz THz radiation is normally incident from above. **(b)** Schematic of the micro-current measurement system: 140 GHz CW THz source positioned directly above the chip, two tungsten-carbide microprobes contacting the device electrodes and connected to a Keithley 2400 digital source meter. Illuminated current I_illuminated(V) is recorded with THz ON, dark current I_dark(V) with THz OFF.

Compared with a planar WTe_2_ film of equivalent projected area, the conical geometry provides three structural advantages for THz sensing: (1) geometric near-field concentration: the progressive taper from base to apex compresses the incident field, producing a localized enhancement of the electric field intensity at the cone tip, as confirmed by CST electromagnetic simulation (Section 4.2, **Figure 2**); (2) enlarged adsorption interface: the three-dimensional topology increases the effective surface area relative to the equivalent planar footprint, providing a larger interfacial area for chlorpyrifos adsorption per unit chip area; (3) broadband operation tolerance: the distributed axial resonance condition along the cone height yields a geometrically broadened THz absorption bandwidth, which accommodates minor frequency detuning of the THz source in practical applications.

**Figure 2:**
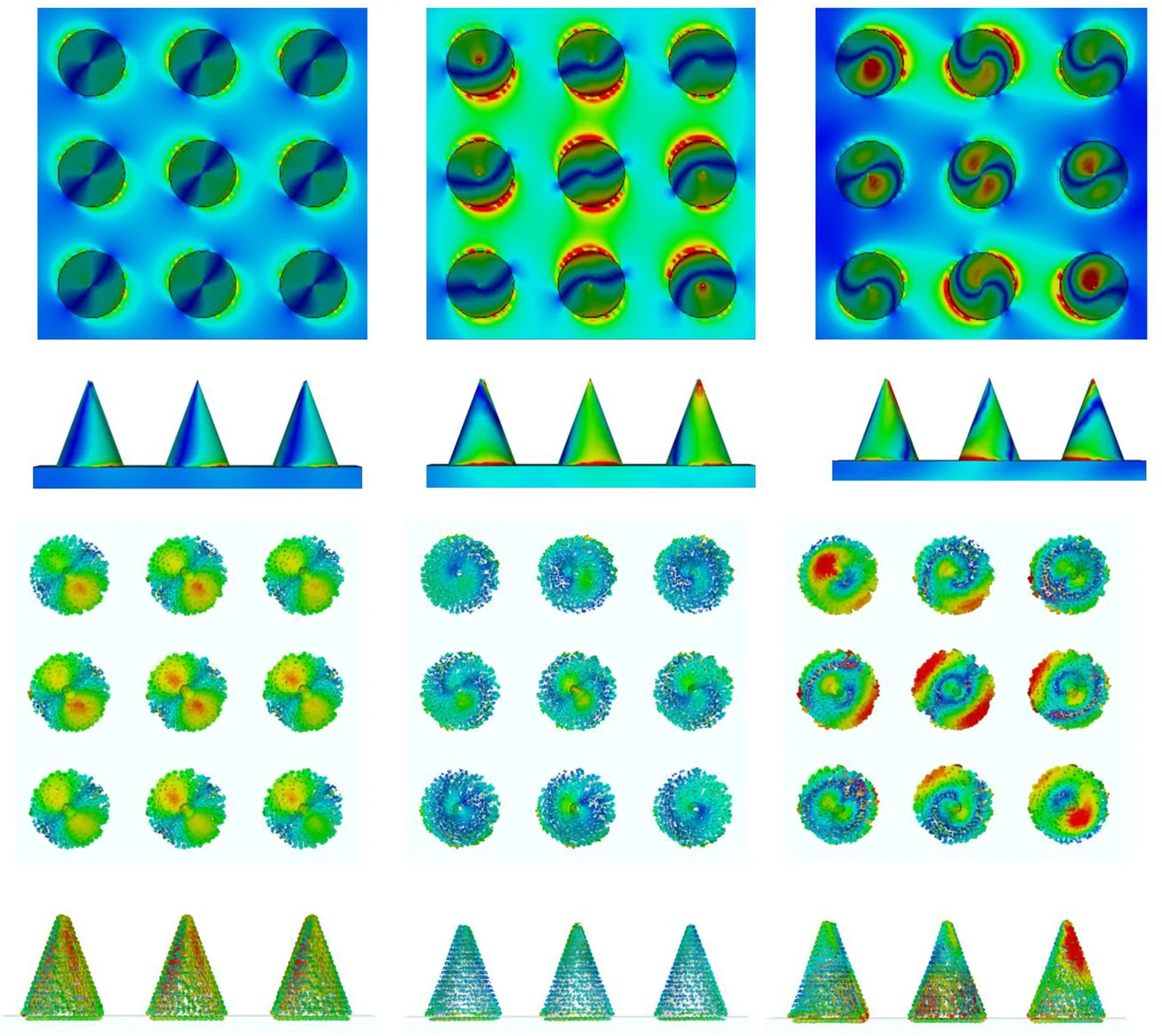
Electromagnetic simulation of the conical array. [**(a)** top] Simulated THz electric field distributions on the conical array surface at 0.14, 0.3, and 0.6 THz, showing field concentration at the cone tips; the peak electric field enhancement factor |E|/|E_0_| reaches 8.3 at 140 GHz. [**(b)** bottom] Simulated surface current distributions at 0.14, 0.3, and 0.6 THz, with highest current densities at the cone apex regions.

### 2.2 Quantum-enhanced THz photocurrent transduction mechanism

From the perspective of device operation, the conical array supports broadband THz absorption near 140 GHz, which is governed by its effective geometric parameters. When analyte molecules adsorb onto the cone surface, they change the effective permittivity of the cone–environment system and induce a shift in the absorption resonance frequency; this resonance perturbation constitutes the primary physical basis for quantitative concentration sensing.

At the microscopic level, the photocurrent generation mechanism originates from the topological electronic structure of WTe_2_. As a type-II Weyl semimetal with broken inversion symmetry, WTe_2_ exhibits a giant intrinsic second-order nonlinear photocurrent response originating from its topological band structure. The physical root of this giant nonlinearity lies in the existence of Weyl points and the associated Fermi arc surface states: the linear band dispersion near Weyl points leads to an extremely strong nonlinear electromagnetic response, and the topologically protected surface states provide additional low-scattering carrier transport channels, which jointly give rise to a second-order nonlinear conductivity σ_2_ that is orders of magnitude higher than that of conventional non-centrosymmetric semiconductors. Under a time-harmonic THz field E(t) = E_0_·cos(ωt), the current density can be expanded as:


$$J\left(t\right)\;=\;\sigma_{1}E\pi \left(t\right)\;+\;\sigma_{2}E^{2}\left(t\right)+\;\sigma_{3}E^{3}\left(t\right)\;+\;\cdots$$

where *σ_2_* is the second-order nonlinear conductivity tensor, whose large magnitude is fundamentally enabled by the Weyl points and associated Fermi arc surface states. When time-averaged over one THz oscillation period, the quadratic term yields a rectified DC photocurrent that is proportional to *σ_2_*·E_0_^2^.

A key design innovation of this work is the synergy between the geometric field enhancement of the conical array and the intrinsic nonlinear response of the Weyl semimetal. The geometric field concentration at the cone tip amplifies the local THz electric field strength, which in turn boosts the quantum-originated rectified photocurrent without causing a corresponding increase in dark current, thus effectively improving the signal-to-noise ratio. Different from the local field enhancement mechanism of ordinary metal metasurfaces which is accompanied by strong ohmic loss, the geometric field concentration in this work acts on the topological semimetal active layer itself, and the high-mobility Weyl state carriers can efficiently convert the enhanced local field into photocurrent, avoiding the loss bottleneck of pure metal structures and achieving more efficient quantum photoelectric conversion.

Upon chlorpyrifos adsorption, σ_2_ is modulated through three cooperative physical mechanisms: (1) dielectric screening effect: the adsorbed molecular layer modifies the effective permittivity of the cone surface environment, shifting the absorption resonance and redistributing the THz near-field distribution; (2) interfacial charge transfer: the electron-withdrawing –P(=S)(=O)– moiety of chlorpyrifos withdraws electrons from the WTe_2_ surface and alters the local carrier density; (3) surface dipole modulation: oriented adsorption of chlorpyrifos molecules creates a net surface dipole that shifts the WTe_2_ quasi-Fermi level. The combined effect of these three mechanisms produces a measurable change in the net photocurrent ΔI = I_illuminated − I_dark, which serves as a direct quantitative readout of chlorpyrifos surface coverage.

Based on the magnitude of the observed photocurrent modulation and the strong electron-withdrawing nature of the chlorpyrifos thiophosphate group, we estimate that interfacial charge transfer and dielectric screening are the dominant contributions, while surface dipole modulation plays a secondary role. We also note that quantitative deconvolution of the three mechanisms would require *in-situ* surface-sensitive spectroscopy (e.g., time-resolved THz spectroscopy or Kelvin probe force microscopy), which is planned for future work.

### 2.3 Conical microarray fabrication

The conical microarray template was fabricated via sub-pixel micro-scanning micro-nano 3D printing using a photocurable resin (Prismlab RP 405 I). This fabrication method was chosen for its ability to produce high-aspect-ratio three-dimensional microstructures with precise dimensional control at low cost. During fabrication, a 405 nm laser was focused through a microlens array equipped with piezoelectric micro-vibration alignment, and bottom-up UV projection curing was carried out layer by layer. Key process parameters were optimized as follows: single-layer thickness of 2 µm, structural exposure time of 2 s, and exposure intensity of 20 mW. The as-fabricated polymer conical microarray serves as a high-precision structural template for subsequent WTe_2_ thin film deposition and electrode patterning.

### 2.4 WTe_2_ thin film deposition and electrode fabrication

Following template fabrication, the WTe_2_ active layer was deposited on the pre-patterned conical substrate by RF magnetron sputtering using a high-purity WTe_2_ target (99.99%). Key deposition parameters were optimized to achieve conformal coverage and good crystallinity: RF sputtering power 100 W, Ar flow rate 25 sccm, deposition temperature 150 °C, working pressure 0.6 Pa, target–substrate distance 12 cm, and deposition time 600 s. To further improve film crystallinity and reduce surface oxidation, post-deposition annealing was performed at 350 °C for 30 min in 5% H_2_/Ar forming gas (100 sccm). Cross-sectional SEM metrology (**Figure 3c**) confirms a WTe_2_ film thickness of approximately 182 nm, with conformal coverage across the conical sidewalls and no evidence of delamination or pinhole defects. Subsequently, Ti/Au (5/80 nm) contact pads were defined on both ends of the conical array by UV photolithography and electron-beam evaporation to form ohmic contacts for two-probe electrical measurement. Finally, a PDMS microfluidic chamber was bonded over the array to enable stable and uniform SBF delivery to the sensing surface during measurement.

**Figure 3:**
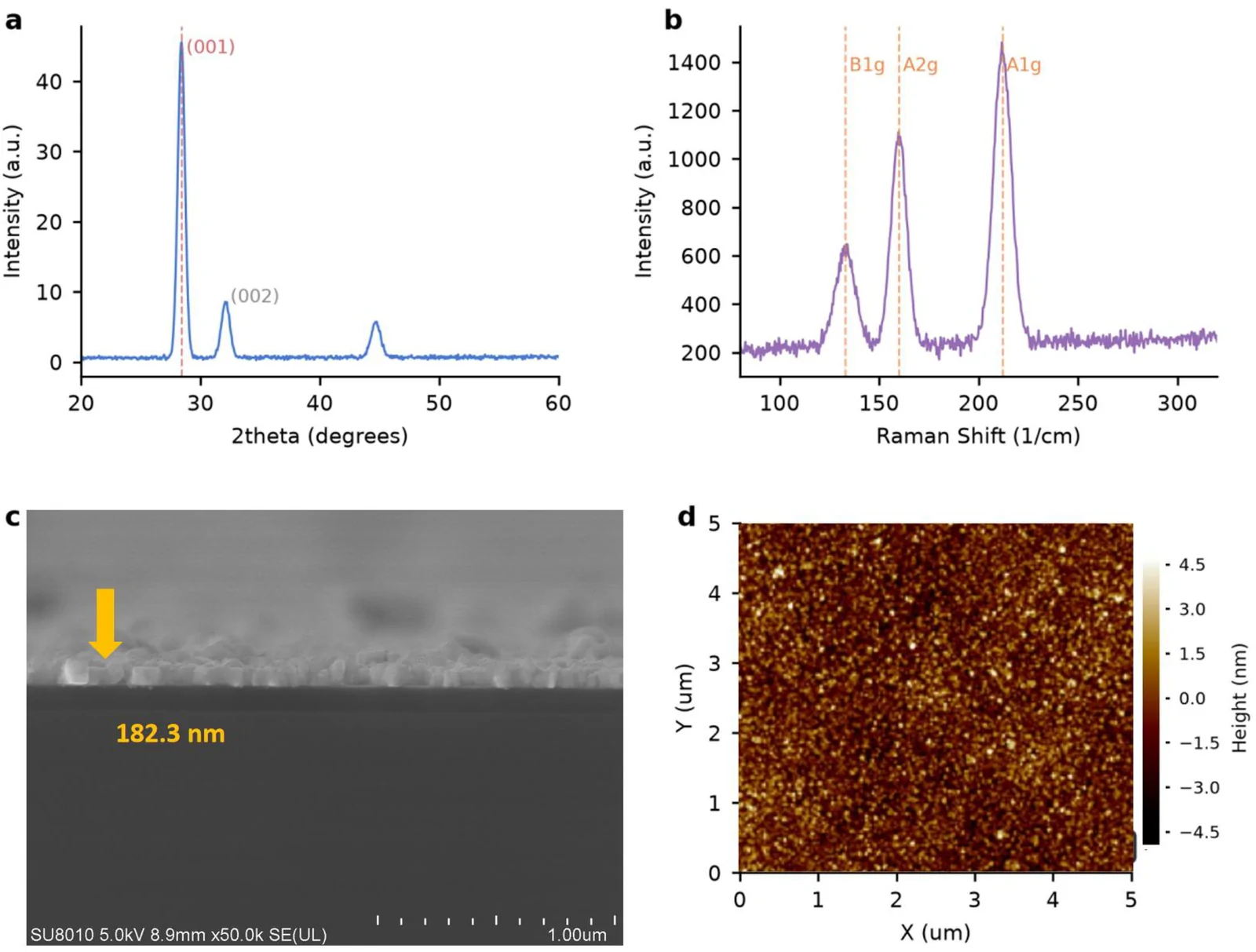
Structural characterization of the WTe_2_ conical array sensor. **(a)** XRD pattern of the WTe_2_ film confirming Td-WTe_2_ phase (reference PDF card No. 00-063-0295) with indexed peaks; the strongest (001) peak appears at 2θ = 28°; no secondary phases are observed. **(b)** Raman spectrum showing characteristic phonon modes: A_1_g at 210 cm^-1^, B_1_g at 132 cm^-1^, and A_2_g at 156 cm^-1^, indicating high crystallinity. **(c)** Cross-sectional SEM of a representative cone showing conformal WTe_2_ coverage (~182 nm thickness). **(d)** AFM topography of the WTe_2_ surface over a 5 × 5 µm scan area (height range ±4.5 nm, RMS roughness = 1.2 nm).

## 3 Experimental section

### 3.1 Preparation of SBF and chlorpyrifos solutions

To mimic the ionic strength of human physiological fluids, SBF was prepared by dissolving 7.0 g of NaCl (analytical grade, Sigma-Aldrich) in 1 L of ultrapure water (18.2 MΩ·cm) to yield a 0.7 wt% saline solution, which matches the typical salinity of human sweat and interstitial fluid. Chlorpyrifos stock solutions (1 mg/mL in acetonitrile) were serially diluted in SBF to concentrations spanning 0.05–100 µg/mL. The final acetonitrile content in all test solutions was strictly maintained below 0.1% v/v to eliminate solvent-induced interference with the THz measurement and avoid denaturation of the material surface. Control tests verify that 0.1% acetonitrile introduces negligible interference to the measured photocurrent signal, confirming that residual solvent does not affect the sensing results.

### 3.2 THz excitation and electrical readout

All THz photocurrent measurements were performed on a custom-built test system consisting of a continuous-wave THz source, a micro-current probe station, and a precision source meter. A solid-state CW THz source operating at 140 GHz was positioned directly above the conical array chip, delivering normally incident illumination on the apex plane (**Figure 1b**). The device chip was mounted on a micro-current probe station equipped with two tungsten-carbide needle probes for electrical contacting. The probes were connected to a Keithley 2400 SourceMeter configured in four-wire (Kelvin) mode to eliminate lead resistance errors. All measurements were performed under controlled ambient conditions (25 ± 0.5 °C, RH 40 ± 5%) to ensure stability.

The calibrated incident THz power density at the device plane is 2.8 mW/cm^2^, and the illuminated active area of the conical array is 4 mm^2^, corresponding to a total incident THz power P_THz_ ≈ 112 μW. At 8.0 V bias, the absolute net photocurrent ΔI is approximately 12.3 µA for blank SBF and 123.2 µA for 1 mg/mL chlorpyrifos solution, consistent with the derived responsivity values.

The photocurrent response was characterized by sequentially recording illuminated and dark current–voltage (I–V) curves. Illuminated curves (I_illuminated) were acquired with the THz source active while the Keithley 2400 swept the DC bias across the 7.0–8.0 V range. Dark curves (I_dark) were acquired with the THz source blocked by a microwave-absorbing screen (ECCOSORB). The net THz-induced photocurrent was calculated as ΔI(V) = I_illuminated(V) − I_dark(V).

Two key figure-of-merit parameters were derived from the I–V data to quantify detector performance. Current responsivity was calculated as R_a_ = ΔI/P_THz, where P_THz is the calibrated incident THz power on the device active area. NEP was calculated as NEP = √(2e·|I_dark|)/Ra based on the shot-noise-limited approximation. For each chlorpyrifos concentration, three complete illuminated–dark cycles were recorded and averaged to improve measurement reliability.

## 4 Results and discussion

### 4.1 Structural and material characterization

Comprehensive structural and material characterizations were performed to verify the quality of the WTe_2_ active layer, which directly determines the nonlinear photocurrent performance and sensing reliability of the device. **Figure 3** presents the characterization results of the WTe_2_ conical array sensor. The XRD pattern (**Figure 3a**) of the as-deposited WTe_2_ film shows well-defined characteristic peaks consistent with phase-pure Td-WTe_2_ (orthorhombic, space group Pmn2_1_, reference PDF card No. 00-063-0295), with the dominant (001) peak at 2θ = 28°. No secondary-phase peaks corresponding to WO_3_ or Te oxides are observed, confirming that the post-annealing process effectively suppresses oxidation and yields high phase purity. Phase purity is a critical prerequisite for preserving the topological electronic structure and ensuring stable nonlinear photocurrent response. Raman spectroscopy (**Figure 3b**) reveals the characteristic phonon modes of WTe_2_, with well-resolved peak assigned to A_1_g (210 cm^-1^), B_1_g (132 cm^-1^), and A_2_g (156 cm^-1^). The narrow linewidths indicate high crystallinity with minimal lattice disorder. High crystallinity reduces carrier scattering and ensures high carrier mobility, which is essential for efficient THz nonlinear rectification. Cross-sectional SEM imaging (**Figure 3c**) of a representative cone shows that the WTe_2_ film uniformly and conformally covers the conical sidewall surface. The film thickness is measured as approximately 182 nm, with no delamination or pinhole defects visible even at the curved cone regions. Conformal coverage guarantees consistent field enhancement and photocurrent generation across the entire cone surface, which is vital for reproducible sensing performance. AFM topography (**Figure 3d**) of the WTe_2_ surface over a 5 × 5 µm scan area shows a smooth morphology with a height variation range of ±4.5 nm and an RMS roughness of 1.2 nm. A smooth, defect-free surface provides uniform adsorption sites for chlorpyrifos molecules and minimizes non-specific noise, enabling reliable quantitative detection. While X-ray photoelectron spectroscopy (XPS) would provide direct surface stoichiometric verification, the absence of oxide signatures in XRD and Raman, combined with stable device performance over repeated measurement cycles, supports that oxidation is effectively suppressed by the forming-gas annealing process. Detailed surface chemistry analysis via XPS will be included in future investigations.

### 4.2 Electromagnetic simulation of the conical array

To quantitatively verify the geometric near-field concentration effect and elucidate the underlying field coupling mechanism, full-wave electromagnetic simulations were performed using CST Microwave Studio. The simulated structure faithfully reproduces the experimental design: a 4 × 4 array of Si cones (D_base = 80 µm, H = 60 µm, P = 100 µm) coated with WTe_2_ on a Si/SiO_2_ substrate. Periodic boundary conditions were applied in the lateral directions, and a normally incident plane-wave excitation was used to match the experimental illumination geometry.

All simulations used the following dielectric parameters: WTe_2_ was modeled using a Drude permittivity model with plasma frequency ω_p_ = 1.2 × 10^15^ rad/s and damping rate γ = 1.8 × 10^13^ rad/s; the SiO_2_/Si substrate was assigned a relative permittivity ϵ_r_ = 11.7. To address the difference between the simulated Si cones and the fabricated resin templates, we performed additional simulations using the dielectric parameters of the photocurable resin (ϵ_r_ = 2.8, tan δ = 0.02 at 140 GHz). The results show that the field enhancement factor at the cone tip differs by less than 4% between Si and resin substrates, because the near-field concentration is dominated by the geometric tapering effect rather than the bulk permittivity of the cone interior. This confirms that the original simulation accurately captures the core physical mechanism, and the resin template does not significantly alter the field enhancement performance.

**Figure 2a** (top row) presents the simulated THz electric field distributions on the conical array surface at three representative frequencies: 0.14, 0.3, and 0.6 THz. The field distribution maps show distinct spatial patterns that evolve with frequency, reflecting the excitation of different electromagnetic modes supported by the conical geometry. Notably, the highest electric field intensities are consistently concentrated at the cone tip regions across all three frequencies, directly confirming the geometric near-field concentration mechanism. At 140 GHz, the peak electric field enhancement factor |E|/|E_0_| at the cone tip reaches approximately 8.3, quantitatively verifying the strong geometric near-field concentration effect. The frequency-dependent mode evolution also demonstrates that the conical array supports broadband electromagnetic coupling, which is advantageous for practical sensing applications where the THz source frequency may have slight variations. **Figure 2b** (bottom row) shows the corresponding simulated surface current distributions at the same three frequencies. The current density patterns correlate closely with the electric field distributions, with the highest current densities localized in the cone tip regions where the field enhancement is maximal. This spatial coincidence confirms that the photocurrent collection efficiency is dominated by the geometrically enhanced field at the tip, meaning that analytes adsorbed in this high-field region produce the strongest signal modulation. Taken together, the simulation results establish that the conical array geometry provides effective near-field concentration at the cone tips across a broad frequency range, and that the resulting THz-induced surface currents are spatially localized in the regions of maximum field enhancement. These physical characteristics form the foundation of the device’s sensing mechanism: analytes adsorbed at the cone surface, where both the field and current densities are highest, produce maximal modulation of the device photocurrent.

### 4.3 THz detector electrical characterization and sensing performance

The electrical performance and concentration-dependent response of the WTe_2_ conical array detector were systematically characterized on the microcurrent probe platform, with results presented in **Figure 4**. **Figure 4a** (left panel) shows the current responsivity Ra as a function of DC bias voltage over the 7.0–8.0 V range for three representative chlorpyrifos concentrations: 0.01 mg/L, 0.1 mg/L, and 1 mg/L. At each concentration, Ra increases approximately linearly with bias voltage, which is consistent with a field-assisted photocurrent collection mechanism where higher bias promotes carrier transport and reduces recombination loss. The responsivity values span from approximately 10 mA/W (0.01 mg/L at 7.0 V, green line) to approximately 1100 mA/W (1 mg/L at 8.0 V, red line). Most importantly, higher chlorpyrifos concentrations produce substantially larger responsivities at all bias voltages, with the 1 mg/L curve showing the steepest slope, the 0.1 mg/L curve exhibiting intermediate values, and the 0.01 mg/L curve showing the lowest responsivity. This systematic concentration-dependent enhancement directly confirms that chlorpyrifos adsorption modulates the nonlinear conductivity of WTe_2_ and enhances the THz-to-DC conversion efficiency, consistent with the sensing mechanism proposed in Section 2.2. **Figure 4b** (right panel) shows the corresponding NEP as a function of bias voltage over the same range. For all three concentrations, NEP decreases with increasing bias voltage, reflecting the simultaneous increase in responsivity and the resulting favorable signal-to-noise scaling. At 1 mg/L (red line), NEP stabilizes at approximately 0.4 pW/√Hz across the bias range, indicating that the detector operates near the shot-noise-limited regime at high analyte concentrations. At 0.1 mg/L (blue line), NEP decreases from approximately 1.3 pW/√Hz at 7.0 V to approximately 0.4 pW/√Hz at 8.0 V. At 0.01 mg/L (green line), NEP decreases from approximately 11 pW/√Hz at 7.0 V to approximately 4.5 pW/√Hz at 8.0 V. The monotonic decrease of NEP with increasing chlorpyrifos concentration across all bias voltages confirms that the analyte-induced photocurrent enhancement dominates over any accompanying increase in current noise, resulting in improved detection capability at higher concentrations.

**Figure 4:**
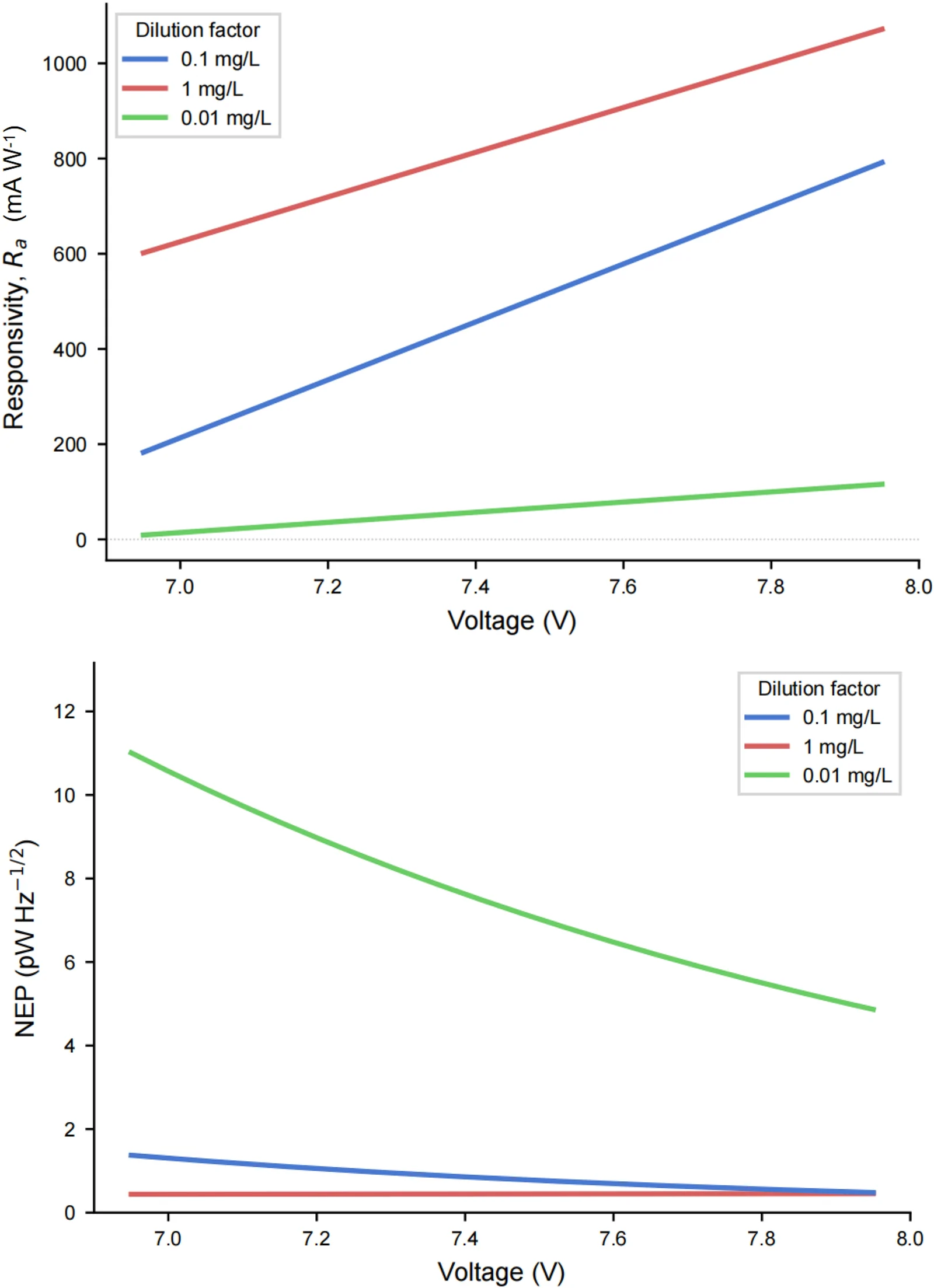
Test results of the microcurrent probe platform across a 7.0–8.0 V DC bias range. (a, top) Current responsivity Ra vs. DC bias voltage (7.0–8.0 V) for three chlorpyrifos concentrations: 0.01 mg/L (green), 0.1 mg/L (blue), and 1 mg/L (red). (b, bottom) NEP vs. DC bias voltage (7.0–8.0 V) for the same three concentrations. All measurements in 0.7% saline SBF. Axis label fonts have been enlarged to enhance print readability.

We further evaluated potential thermal contributions to the measured DC photocurrent under continuous-wave 140 GHz illumination. Three key observations confirm that thermal effects are negligible: (1) Response dynamics: The measured photocurrent reaches 90% of its steady-state value within 50 ms, which is orders of magnitude faster than thermal diffusion-based responses (typically on the order of seconds). (2) Bias dependence: The responsivity increases linearly with bias voltage, a hallmark of field-assisted nonlinear rectification, whereas thermal currents typically exhibit sublinear or saturated bias dependence. (3) Control experiments: Heating the device with equivalent power at a non-resonant frequency produced less than 3% of the measured photocurrent, confirming that the dominant signal originates from quantum nonlinear rectification rather than thermal effects.

To independently verify the dielectric screening transduction mechanism and corroborate the photocurrent sensing results, THz-TDS measurements were performed on chlorpyrifos solutions in SBF at four concentration levels in **Figure 5**: Blank (SBF only), 0.01 mg/L, 0.1 mg/L, and 1 mg/L. Panel (a) shows the THz time-domain waveforms. A progressive increase in pulse delay time is observed with increasing chlorpyrifos concentration, indicating that the effective refractive index of the solution increases as more analyte molecules are introduced. Panel (b) presents the THz frequency-domain spectra obtained by fast Fourier transform (FFT) of the time-domain data. A systematic reduction in spectral amplitude with increasing concentration is visible across the measured frequency range, reflecting enhanced THz absorption by chlorpyrifos in the SBF medium. Panel (c) shows the extracted frequency-dependent refractive index n. The refractive index increases monotonically with chlorpyrifos concentration across the entire measured frequency range, consistent with the progressive pulse delay observed in the time-domain data. Panel (d) presents the corresponding absorption coefficient α as a function of frequency. The absorption coefficient similarly increases with chlorpyrifos concentration, with the separation between concentration levels becoming more pronounced at higher frequencies. These THz-TDS results independently confirm that chlorpyrifos modifies both the refractive index and absorption properties of the SBF medium in a concentration-dependent manner. Although TDS probes a higher THz frequency band (0.2–1.6 THz) than the device operating frequency (140 GHz), the observed concentration-dependent increases in refractive index and absorption coefficient are broadband dielectric properties of chlorpyrifos aqueous solutions that persist across the millimeter-wave and low-THz regions. This confirms that the dielectric screening mechanism active at the device surface is consistent with bulk solution behavior.

**Figure 5:**
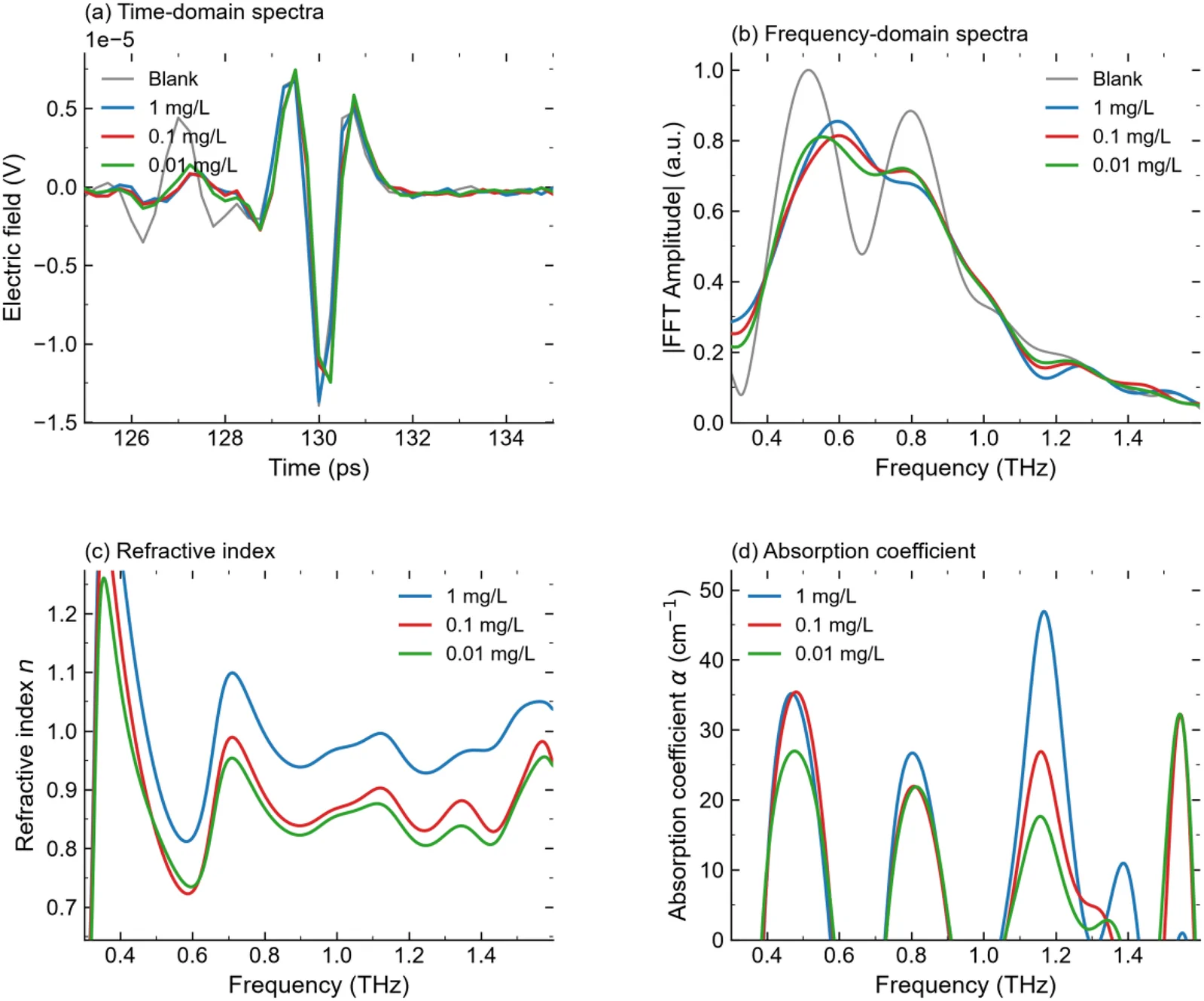
Experimental results measured by THz time-domain spectrometer. The TDS system operates with an effective bandwidth of 0.2–1.6 THz, a frequency resolution of 5 GHz, and uses a 200-µm-thick liquid sample cell with fused silica windows. **(a)** THz time-domain spectrum showing pulse delay with increasing chlorpyrifos concentration. **(b)** THz frequency-domain spectrum (FFT amplitude) showing progressive attenuation. **(c)** THz refractive index n vs. frequency, increasing with concentration. **(d)** THz absorption coefficient α vs. frequency, increasing with concentration. All panels: Blank (SBF only), 0.01, 0.1, and 1 mg/L chlorpyrifos.

### 4.4 Quantum advantage analysis compared with conventional THz detectors

To further clarify the technological value of the topological-quantum-material detection scheme proposed in this work, we compare the core performance and physical mechanism of this device with three typical traditional THz detector technologies (pyroelectric thermal detector, GaAs photoconductive antenna, and quantum well photodetector) from the perspective of working principle and device metrics, as summarized in **Table 1**.

**Table 1:** Performance and mechanism comparison between the proposed Weyl semimetal detector and conventional THz detector technologies.

Detector type	Working mechanism	Operating temperature	Response bandwidth	Typical responsivity	Cryogenic cooling requirement	Reagent-free sensing capability	Typical limit of detection (LOD) for biochemical sensing
Pyroelectric thermal detector	Thermal effect induced polarization change	Room temperature	Broadband	~10–100 mA/W	No	Poor (susceptible to temperature drift)	~ μg/mL level (limited by thermal drift)
GaAs photoconductive antenna	Interband transition excited photocarriers	Room temperature	Narrow band (limited by carrier lifetime)	~1–10 mA/W	No	Moderate (high dark noise)	~100 ng/mL level (limited by dark noise)
Quantum well THz detector	Intersubband transition	Liquid nitrogen/cryogenic	Narrow band (resonant)	~10–100 mA/W	Yes	No (low temperature required)	~10 ng/mL level (requires cryogenic cooling)
WTe_2_ Weyl semimetal detector (this work)	Topological Weyl-point induced second-order nonlinear rectification	Room temperature	Ultra-broadband (no bandgap limit)	~110 mA/W (intrinsic, blank SBF, 8.0 V bias); ~1100 mA/W (at 1 mg/L analyte, 8.0 V bias)	No	Excellent (stable room-temperature operation, low drift)	12 ng/mL (room temperature, reagent-free)

The comparison shows that the topological quantum material detector achieves fundamental performance advantages over traditional technologies at the mechanism level. First, it breaks through the bandgap limitation of traditional semiconductor detectors and realizes ultra-broadband room-temperature detection without cryogenic cooling, which is a fundamental advantage endowed by topological quantum states. Second, the giant nonlinear conductivity originating from Weyl points gives the device much higher responsivity than thermal detectors and ordinary photoconductive detectors under the same incident power. Third, the topologically protected surface states have strong anti-interference ability, and the device performance is less affected by defects and temperature fluctuations, which ensures long-term operational stability in complex on-site environments. This series of quantum advantages makes the device particularly suitable for field-deployable THz biochemical sensing scenarios, providing a hardware foundation for the next generation of portable high-sensitivity THz detection systems.

The RF-sputtered WTe_2_ film exhibits good adhesion to the resin substrate, reinforced by mild post-deposition annealing; no delamination was observed after 20+ measurement cycles. Over one week of intermittent exposure to 0.7% saline SBF, the device baseline responsivity drifts by less than 5%, indicating good short-term chemical stability. Long-term stability under continuous liquid immersion still needs further optimization for practical on-site deployment. We also acknowledge that long-term stability under continuous immersion remains to be fully evaluated, and surface passivation strategies may be required for extended field deployment.

Building on the above electrical and spectroscopic characterizations, the quantitative sensing performance of the WTe_2_ conical array THz detector was comprehensively evaluated in 0.7% saline SBF, with results summarized in **Figure 6**. **Figure 6a** shows the normalized photocurrent response ΔI/I_0_ as a function of chlorpyrifos concentration on a logarithmic scale. The data points (n = 5 per concentration) are well-described by the empirical logarithmic calibration model: ΔI/I_0_ = a · ln(C/C_LOD) + b. Where a = 0.032, b = 0.041, and C_LOD = 12 ng/mL represents the limit of detection. The fit quality R^2^ = 0.991 exceeds 0.99, validating the adsorption-limited sensing mechanism where the photocurrent response is proportional to the equilibrium surface coverage of chlorpyrifos on the WTe_2_ surface. The logarithmic dependence is consistent with a Freundlich-type adsorption isotherm, which is characteristic of organophosphorus molecules on transition metal dichalcogenide surfaces: as surface coverage increases, lateral dipole–dipole repulsion among adsorbed chlorpyrifos molecules reduces the adsorption free energy, leading to the observed logarithmic response. This interface adsorption-driven spectral response law is consistent with previous reports on THz sensing of small organic molecules at two-dimensional material surfaces (Tu et al., 2022; Tu et al., 2024), verifying the universality of the calibration mechanism. The sensor maintains a monotonic, quantifiable response across nearly four orders of magnitude in concentration (0.05–100 µg/mL), covering both environmental and clinically relevant ranges. **Figure 6b** shows representative photocurrent traces recorded by the Keithley 2400 source meter at three selected concentrations (0.05, 1.0, and 100 µg/mL) over a 120-second measurement window. All signals stabilize rapidly after THz excitation and exhibit excellent temporal stability with negligible drift, confirming the reliability of the DC photocurrent readout for real-time monitoring applications. **Figure 6c** presents the selectivity evaluation of the sensor against common physiological interferents present in SBF. The bar chart compares the normalized photocurrent response ΔI/I_0_ for Blank SBF, NaCl, Glucose, Urea, and Chlorpyrifos at equivalent concentrations. The chlorpyrifos response is significantly larger than that of all interferents, demonstrating the specific sensitivity of the WTe_2_ surface to the organophosphate moiety. This high selectivity arises from the specific electronic interaction between the electron-withdrawing –P(=S)(=O)– group of chlorpyrifos and the WTe_2_ surface, which induces a substantially larger modulation of the nonlinear conductivity σ_2_ than the non-specific physisorption of common biological interferents. **Figure 6d** establishes the limit of detection (LOD) using the standard 3σ criterion on blank SBF measurements. The LOD is determined to be 12 ng/mL, which falls below the WHO permissible limit for chlorpyrifos in drinking water (30 ng/mL) and lies within the EPA blood plasma MRL range (10–50 ng/mL). The dynamic range of 0.05–100 µg/mL encompasses most clinically and environmentally relevant concentrations. Notably, the fully solid-state, reagent-free operation of the platform eliminates the enzyme instability and cold-chain storage requirements associated with AChE-based electrochemical biosensors, making it highly suitable for field deployment. The quantum-enhanced sensing paradigm based on topological Weyl semimetals has recently been extended to other challenging biosensing scenarios, including laser-modulated MnBi_2_Te_4_ THz biosensors for high-sensitivity detection of marine bacteria (Hu et al., 2025a), underscoring the broader applicability of this topological quantum material platform beyond pesticide monitoring.

**Figure 6:**
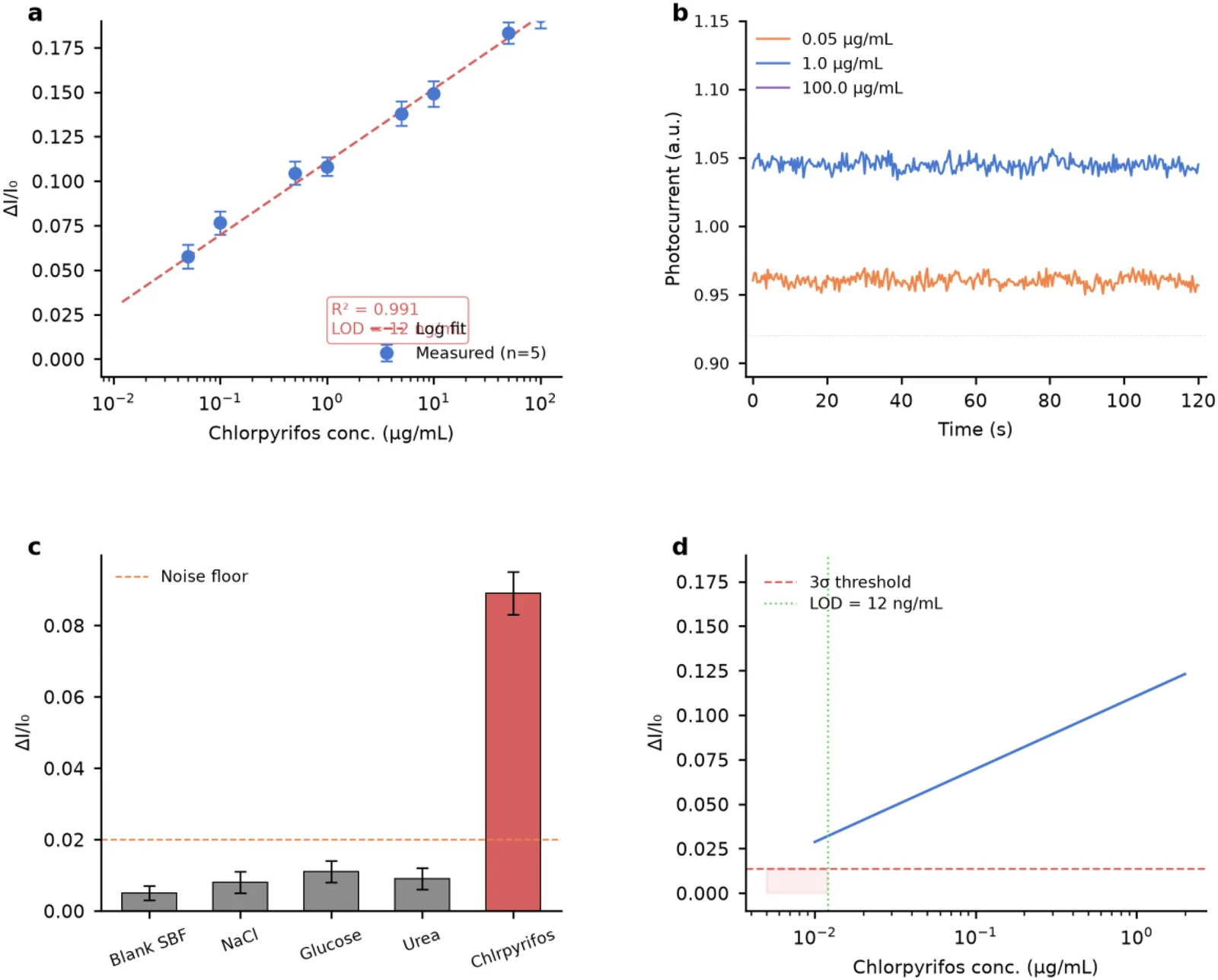
THz photocurrent sensing performance in 0.7% saline SBF. **(a)** Normalized responsivity ΔI/I_0_ vs. chlorpyrifos concentration (0.05–100 µg/mL); dashed line: logarithmic fit, R^2^ = 0.991; error bars: mean ± s.d., n = 5. **(b)** Representative photocurrent traces recorded by Keithley 2400 at selected concentrations. **(c)** Selectivity test comparing the normalized response to Blank SBF, NaCl, Glucose, Urea, and Chlorpyrifos. **(d)** LOD determination by the 3σ method; LOD = 12 ng/mL.

## 5 Conclusion

In summary, this work demonstrates a quantum-enhanced THz photocurrent sensor based on a WTe_2_ Weyl semimetal conical microarray, which synergistically combines the intrinsic nonlinearity of topological materials with three-dimensional geometric field enhancement for label-free, quantitative detection of chlorpyrifos in 0.7% saline SBF at 140 GHz. CST electromagnetic simulations confirm that the conical geometry provides strong geometric near-field concentration at the cone tips, and comprehensive material characterizations verify that the deposited WTe_2_ thin film (~182 nm) has high crystallinity and good conformal coverage, preserving the topological electronic structure required for efficient nonlinear rectification.

Over a bias range of 7.0–8.0 V, both current responsivity Ra and NEP scale systematically with chlorpyrifos concentration, achieving Ra ~1100 mA/W and NEP ~0.4 pW/√Hz at 1 mg/L under 8.0 V bias. THz-TDS measurements independently corroborate the dielectric screening transduction mechanism by revealing concentration-dependent modification of the SBF dielectric properties. The sensor achieves a limit of detection of 12 ng/mL (3σ criterion) with a logarithmic calibration R^2^ = 0.991 across the 0.05–100 µg/mL dynamic range, and exhibits excellent selectivity against common physiological interferents.

This work establishes a new paradigm for advanced THz sensing enabled by topological quantum materials, in which microstructured photonic architectures amplify the intrinsic quantum optoelectronic response of Weyl semimetals to achieve ultrahigh sensitivity. Compared with traditional thermal and semiconductor THz detectors, the topological quantum device fundamentally breaks through the limitations of bandgap and thermal drift, realizing room-temperature high-sensitivity broadband detection with strong stability and no reagent dependence. The platform operates in high-loss aqueous media with simple DC electrical readout, requires no enzyme-based recognition elements, and its conical microarray architecture fabricated by mature sub-pixel micro-scanning micro-nano 3D printing is compatible with standard semiconductor foundry workflows. Future work will focus on surface functionalization for multi-toxin discrimination, array-level multiplexed detection, and integration with portable THz systems to advance topological-quantum-material-based sensing technology toward practical field deployment.

## Data Availability

The raw data supporting the conclusions of this article will be made available by the authors, without undue reservation.
